# Outlooks on using a mobile health intervention for supportive pain management for children and adolescents with cancer: a qualitative study

**DOI:** 10.1186/s12912-023-01461-z

**Published:** 2023-09-04

**Authors:** Dina Madi, Myrna Abi Abdallah Doumit, Mohammad Hallal, Maya M. Moubarak

**Affiliations:** 1 Hariri School of Nursing, American University of Beirut, Beirut, Lebanon; 2 Biomedical Engineering Program, Maroun Semaan Faculty of Engineering and Architecture, Faculty of Medicine, American University of Beirut, Beirut, Lebanon

**Keywords:** mHealth app, Children, Cancer, Pain Management, Social Support

## Abstract

**Background:**

Considerable improvements in the prognosis of pediatric cancer patients have been achieved over recent decades due to advances in treatment. Nevertheless, as the most common and distressing health issue for pediatrics with cancer, cancer-related pain is still a significant hurdle that impedes patients’ journey to recovery, compromises their quality of life, and delays the positive outcome and effectiveness of their treatments.

**Purpose:**

Taking into consideration that acceptability studies are imperative for the design, evaluation, and implementation of healthcare interventions, this study aims to explore pediatric oncology patients’ readiness to use a mobile health application that emphasizes social assistance and peer support in addition to conventional pain management methods.

**Design and methods:**

This study followed the Qualitative description approach. Twelve participants were chosen based on purposive sampling and maximum variation sampling. Interviews were analyzed using the conventional content analysis.

**Results:**

Analysis of the interviews revealed four major categories: (A) The need for connectedness; (B) An innovative way to connect yet fearful; (C) A 3D approach; (D) Fears of the unfamiliar.

**Conclusions:**

This study is the first in Lebanon and the region to undertake an initiative towards introducing technology for pain assessment and management of children with cancer through a dedicated digital platform. The study results attested to the acceptability and potential utilization of this platform by children with cancer.

**Practice implications:**

Nurses need to be trained to play an essential role in teaching children with cancer about the significance of social support and assisting them to establish their social support network. Children with cancer are encouraged to voice out their need for help. Our proposed application can create an enabling environment to harness the power of social support and provide children with cancer the opportunity to connect on a deeper level in a supportive and pity-free space.

## Background

Pain is a significant health issue that is considered the most common and distressing symptom for cancer patients 1–6]. Considerable improvements in the prognosis of children and adolescents with cancer were achieved over the past decades due to advances in treatment protocols [7–10]. Yet, cancer-related pain is still a significant hurdle, as it impedes cancer patients’ journey to recovery and compromises their quality of life by negatively affecting their physiological and psychological states [11, 12]. Moreover, it delays the positive outcome and effectiveness of their treatment [13, 3]. Despite access to opioids [14–16] and the available strategies for managing cancer-related pain [17–21], pain undertreatment is still widely reported among pediatric cancer patients [22, 23] to the extent that up to 50% of patients experience inadequately controlled cancer-related pain [24]. In Lebanon a recent study by Finianos et al., (2021) reported that since 2010 only eight studies were published about pain in the pediatric population which denotes a scarcity in this area of research. Also, the authors highlighted the need to improve management of pain in young Lebanese people in terms of opioids availability and better training of professionals which could help to positively change the situation of the treatment of children and adolescents with pain in the country.

### Psychosocial aspect

In addition to physical management, children and adolescents resort to using psychosocial management and interpersonal resources to manage their pain [25]. Indeed, children and adolescents with cancer have distinct psychosocial needs compared to their adult counterparts [26]. Moreover, it has been shown that childhood cancer survivors with perceived social isolation and loneliness reported poorer physical functioning, higher pain intolerance, fatigue, anxiety, and depression compared to those who did not experience loneliness or to cancer-free control groups [27]. This supports the idea that the health of an individual is influenced by the level of connectedness to others, which in turn is achieved through social support resulting in a lower degree of perceived social isolation, higher levels of physical and mental well-being, as well as adherence to healthy behaviors [27, 28].

In this context, capitalizing on social resources and facilitating communication [29] can serve as a proxy method of pain management, as well as a tool to improve adherence to therapeutic regimens, and to promote a better sense of well-being in children and adolescents with cancer [30–34]. Research studies showed that children, and adolescents with cancer who received social support from their family, friends, peer cancer patients or others displayed improvement in their quality of life [35]-[38] and a significant decrease in the stress and burden experienced by parents [35, 39–41]. These results suggest the presence of concentric circles of social support: an innermost circle represents parents and close family members with whom the patient has face-to-face interactions, and outer circles encompassing friends, caregivers, and peer cancer patients.

Another equally important aspect of pain management is pain reporting. Increased patient self-reporting of pain and symptom monitoring has been shown to enhance patients’ quality of life, reduce unexpected usage of health care services, and improve adherence to anti-neoplastic therapy [42]. Paper-based self-reporting is one of the most extensively used pain evaluation approaches [42]. However, it relies on the patient’s recollection of prior experiences leading to reporting errors and biases [43]. On the other hand, electronically-based pain evaluation methods, are a superior way to obtain patient pain data in an out-of-clinic setting [1, 44, 45] and can improve the assessment of symptoms using digitized scales [29]. These methods can also increase health literacy among patients and their caregivers [45] through, for example, instant access to information about cancer prevention or screening and treatment options via mobile applications, thus encouraging patients to become more active in managing their medical care. Combining pain-management tools with social support, therefore, introduces a new value proposition whereby the integration of these approaches could be beneficial to patients experiencing pain [46].

### Mobile Health

Mobile health (mHealth) applications can be geared towards achieving different outcomes such as symptoms monitoring [47], sharing experiences [48], pain assessment [49], monitoring of treatment related symptoms (C-SCAT), and documenting self-management strategies [31]. Nevertheless, relying on digital and electronic means for healthcare purposes is fairly recent, and research investigating the components and efficacy of pain-related mobile health applications is an emerging field [1, 50]. Among some of the positive features of mHealth applications identified in the literature, recent applications, such as the Pain Guard app, helped to engage hospital professionals with discharged cancer patients and proved to be effective for managing cancer pain and enhancing patient quality of life (QoL) [51]. In addition, the reminder feature found in many mHealth applications has proven to be beneficial as it promotes high adherence rates and compliance to reporting symptoms [47, 49, 52]. It is no surprise, then, that the use of mobile applications for pain management is gaining popularity amongst health care professionals [45, 53, 54]. On the other hand, there seems to be a lack of abundance of mHealth applications that are truly effective and usable. For instance, among 295 commercially available cancer smartphone applications identified and assessed by Bender et al. (2013), only a few were found to have real practical benefits. Moreover, adherence and reported satisfaction of using pain management applications were subject to perceived ease-of-use [25]. Importantly, almost none of these available applications capitalizes on the power of peer-centric or guided social support as a pain management tool. Indeed, restricted access to social support is a well-identified critical gap in current pain management approaches [47, 49, 55], especially that connecting to support groups is not a typical feature of most medication management apps [56] .

As for children and adolescents with cancer pain, little is known about the factors that make smartphone technology acceptable for this population [57, 58]. The few available studies, however, have highlighted that perceived ease-of-use [25], the ability to track progress [46], communicating with healthcare providers [59] and other patients with a similar condition [27] were important determinants. Taking into consideration that acceptability studies are imperative for the design, evaluation, and implementation of healthcare interventions [60] we aim to investigate the acceptability of a mobile health intervention for supportive pain management centered around social support within the Lebanese children and adolescent cancer patients community. Furthermore, based on the need for evidence supporting the benefits of use of mHealth applications and investigating the impact of social support, we seek to evaluate the perceived benefits of developing a new digital platform that addresses the gaps in currently available mobile applications while emphasizing the provision of social assistance and peer support.

## Method

### Design

This study followed the Qualitative Description (QD) which is a categorization used in qualitative research for studies that are descriptive in nature, mainly for exploring health care and nursing-related phenomena [61]. QD is the method of choice when focusing on discovering the who, what, and where of events or experiences, gaining insights from informants regarding a poorly understood phenomenon or getting a straight description of a phenomenon [62, 63]. For the purpose of this study, the QD design was followed since we were seeking to evaluate the extent of acceptability of a new, never before explored within the Lebanese culture, approach to pain assessment and management for Lebanese children and adolescents.

### Participants

Twelve participants were chosen based on purposive sampling. The number of participants was determined by reaching a point of data saturation whereby no new ideas were being offered in the newly collected information [64]. Saturation was reached with the tenth participant; however, two additional interviews were conducted to make sure that no new ideas would emerge. Children and adolescents were invited to participate in the study if they were Lebanese, between 9 and 17 years old, diagnosed with any type of cancer; and on treatment during the time of the study. Children and adolescents were excluded from the study if they were terminally ill; and had a pre-existing mental/cognitive disorder, or hearing/speech problem. The maximum variation sampling approach was followed in this study which is the most useful sampling for the naturalistic approach [65]. The variation was based on age, gender, education, socio-cultural and religious background.

### Ethical considerations

Approvals for studying human participants were obtained ahead of conducting the study from the Institutional Review Boards (IRB) of the American University of Beirut (# SBS-2020-0016), the American University of Beirut Medical-Center (AUBMC) director and the director of the Children Cancer Center of Lebanon (CCCL) at AUBMC. Established procedures for protecting confidentiality were strictly followed. All participants read and signed an informed assent form while their parents signed an informed consent form. Additionally, participants chose a pseudonym to maintain anonymity. At the first meeting and all subsequent interactions, each participant was reminded that his/her participation was voluntary and that at any time he/she could decline or withdraw from the study without any obligation. It is worth noting that one eligible candidate whom we approached did not accept to take part in the study and one participant later withdrew from the study because she could not tolerate the interview. Both were between 9 and 10 years old and their background matches the group of participants that were involved with the study. All interviews were coded so that only the principal investigator (PI) and the co-investigator (Co-PI) could identify the participants. The code list and the original tapes were securely archived in a locked file cabinet in the PI’s office for a period of three years. Given that the interviews were conducted in Arabic, then translated and typed in English and back-translated to Arabic the transcriptionist and the translators signed a confidentiality agreement as well.

### Recruitment of participants

Children and adolescents with cancer receiving care at the Children Cancer Center at the American University of Beirut, along with their parents, were approached by the nurse manager to inquire about their willingness to participate in the study. It is worth noting that this center receives patients from the MENA region. After ensuring the consent of parents and the assent of the child, candidate participants were then contacted by the PI to confirm their agreement to participate in the study and to set a convenient appointment. This approach fits well the tenets of sample selection for a qualitative study [66, 67]. All face-to-face interviews took place at the CCCL at AUBMC in a quiet environment as per the participants’ request.

### Data collection

Data were collected between June, 2021 and September, 2021, using semi structured in-depth interviews conducted by the PI. In total, 12 participants [mean (M) age = 13.66 years]; five girls and seven boys were invited to participate in this study. The participants’ ages ranged from eleven years to seventeen years Demographic variables and diagnoses (e.g., age, gender, educational level, socioeconomic status, place of living) for all participants are presented in Table 1. All participants (100%) were under chemotherapy treatment and 58% had metastasis to other organs. These children and adolescents had been on treatment from six months to two years and 10 months before they were interviewed.

**Table 1:** Demographic characteristics of study participants (n = 12)

*Characteristic*	*Frequency*	*Percentage*
Age (year)		
Mean (SD)	13.6 (2.26)	
11–13	7	58%
14–17	5	42%
Gender		
Female	5	42%
Male	7	58%
Educational Level		
Grade 3–5	4	33%
Grade 6–8	6	50%
Grade 8 and above	2	17%
Socioeconomic Status		
Low	8	67%
Middle	3	25%
High	1	8%
Place of Living		
Rural	11	90%
Urban	1	10%
Cancer Diagnosis and Primary Site		
Glioma	2	17%
Leukemia	5	42%
Medulloblastoma	1	8%
Osteaosarcoma	3	25%
Osteocytoma	1	8%
Remission		
Yes	5	42%
No	7	58%
Metastasis		
Yes	5	58%
No	7	42%
Cancer Treatment Received		
Chemotherapy	12	100%
Treatment Duration		
0.5–1.5 years	6	50%
2–3 years	6	50%
Siblings		
Yes	12	100%
Device		
Smartphone	12	100%
Laptop	4	33%

Participants were interviewed twice. The purpose of the second interview was to validate the preliminary analysis of the first interview. The time lapse between the first and second interviews was approximately two weeks. The first interview lasted 50 to 60 min while the second interview lasted 30 to 40 min. All participants tolerated both interviews without any reported physical or psychological disturbances. Demographic data was collected from the participant at the beginning of the first interview. To minimize participant’s socially pleasing answers [65], the PI, at the beginning of each interview, clarified that she was only concerned about the individual’s thoughts and feelings regarding the willingness of the participant to connect with others using an application and the demonstrated acceptability of utilizing such an application. The participant was asked to answer the following questions “How do you feel about reporting your pain and getting support from adolescents with cancer like you through a smartphone app?” OR “When you are in pain what are the things you do to deal with your pain other than taking medications?” Participants were asked to provide examples that would help to describe their ideas using probing such as, “Please tell more about it”, “What does that mean to you?”, “Is it possible to give an example?”, “Describe to me what that was like for you” were used to elicit further explanations. It is worth mentioning that no two interviews were expected to be precisely the same. During each interview, the participant was the major speaker, and the investigator was mainly a listener and facilitator. The interviews were tape-recorded, transcribed and translated to English, and back-translated to Arabic The PI also recorded field observation notes.

### Data analysis

Interviews were analyzed using the Conventional Content Analysis (CCA) as recommended by Hsieh and Shannon (2005). The PI and Co-PI, each on her own, read all data repeatedly to achieve immersion and obtain a sense of the whole. Then, data were read word by word to derive children and adolescents’ codes by first highlighting the exact words from the text that appear to capture key thoughts or concepts. Next, the PI and Co-PI approached the text by making notes of their first impressions, thoughts, and initial analysis. As this process continued, labels for codes emerged that are reflective of more than one key thought. These came directly from the text and then became the initial coding scheme. Codes were then sorted into categories based on how they are related and linked. These emergent categories were used to organize and group codes into meaningful clusters, then exemplars for each code and category were identified from the data. The PI and Co-PI, who are bilingual (English, Arabic), made sure to maintain the conceptual equivalence of what participants said during interviews by reviewing the translation and back translation done by translators [68, 69]. PI and CO-PI did not encounter any difficulties since the English version reflected a technically and conceptually accurate translated communication of the interviews made by the participants. Data collection and analysis were done concurrently throughout the study.

### Trustworthiness of the study

A weakness of CCA is its potential to inadequately seize the context of the phenomena under investigation and thus fail to capture main codes during analysis [70]. To improve threats to validity, Creswell (2013) endorses that all qualitative research includes at least two forms of verification strategies into its methodology; in the current study we included at least five: researcher reflexivity, investigator triangulation, data triangulation, peer-debriefing and thick-rich description.

## Findings

This first study, within the Lebanese culture, assessing the suitability of a tool to connect with others and to assess and document cancer related pain yielded reflective results. All participants expressed their need and willingness to connect to other patients with similar condition to share their cancer experiences. These results are aligned with findings from previous studies by Doumit et al. (2007) where adult cancer patients reported the need to discuss their conditions with cancer patients, to be better understood, and not to be pitied. Analysis of the interviews revealed four major categories: (A) The need for connectedness; (B) An innovative way to connect yet fearful; (C) A 3D approach; (D) Fears of the unfamiliar (Table 2).

**Table 2:** Conventional content analysis codes and categories with definitions

Category	Definition	Code	
The need for connectedness	The important feeling of relating to others	A person willing to listen	
		A person willing to understand and has a caring attitude	
		Connecting with another person sharing similar experience	
An innovative way to connect yet fearful	A new approach for feeling close to others	A mean for sharing pain	
		Agreeable idea	
		Fearful about confidentiality, bullying and the unknown	
A 3 D Approach	The need to mimic reality while meeting others	Living with friends in cyberspace	
		Availability of the other at all times in cyberspace	
Fear of the unfamiliar	Uncertainty	Human factor barriers: Disease related shame, language…	
		Technology factor: Size of the application	

### The need for connectedness

All participants expressed the need to connect. They all emphasized the importance of having someone who is willing and able to hear and understand their needs. At the beginning of the interviews almost all participants identified family members, mainly their mothers, as the person of choice to connect with. Later, with probing, they started to identify other individuals to connect with such as friends they had made at the cancer center who were suffering the same pain and who could understand and interpret their feelings. This idea of connectedness with others who could understand what they were going through was key for accepting the application.

Zouzou, a 16-year-old adolescent said:

*Since our immunity would be low, having someone to talk to, someone who lived the same experience and is passing through the same issues would help a lot especially upon admission.*

Along the same idea Batman, an 11-year-old boy mentioned:

*When I am in pain I want to speak and report to friends who are with me in the center here…. Talking to them would be fun and I can forget my pain.*

Queen, a 12-years-old girl who does not have friends at the center said:

*The application is a good idea….I do not know any patient here in the center, but I would like to have because these patients know my pain and this app will help me connect with them*.

Along the same line, Ronaldo, a 17-years-old boy stated:

*The idea of this application is nice…….I will report my pain to my friends in the center using this app, anyway these are the ones I usually tell them about my pain since they have the same experience and they know how it feels.*

Hamada, a 16-year-old adolescent said:

*The app is a nice idea, it is helpful to connect with others especially when you are in the hospital feeling lonely and have no one to talk to you and understand what you are going through*.

Ronaldo, a 17-year-old adolescent associated being connected on the app with his eagerness to come to the center:

“*The app will help you connect and make new friends, this way one will be encouraged and eager to come to the center to meet these new friends. You know, children hate to come to the center and if you ask most of them if they are coming willingly, they will say no because they feel bored as most do not have friends here. But, if they make friends, they will be eager to come and meet the new friends, connect directly with them, and have fun*”.

### An innovative way to connect yet fearful

All participants willingly accepted the concept of using an application to share their pain and concerns, and found the idea very appealing and agreeable although it is new to the Lebanese culture. They, however, expressed concerns about how confidentiality would be managed in such applications.

Bob, a 12-year-old boy stated:

*I usually talk about my pain to my mother and sometimes to my father, I do not like to talk about my pain to others. I do not have friends in the center, I had one but he passed away. But this application is a good idea, because I can use it to help others through storytelling and jokes. I like jokes and use them to relieve my own pain.*

Princess, a 13-year-old girl who feels at ease talking about her pain to other patients with the same condition reported that:

*The app is a nice idea, especially if we can talk to other patients who suffer from a similar condition and who can understand what we are feeling. This way I will feel comfortable and not ashamed that I am different from other healthy people*.

Princess added that:

*but I would like the app to be private, I mean that I want my information; my name, my diagnosis, what is happening with me to be confidential. Let it be a way to talk about pain with other patients who have the same experience and can understand my feelings.*

Yaldiz, a 12-year-old girl who was concerned about how other perceived her condition:

*It would be great if the app can be used with a camera that would allow you to see other patients talking about your pain, they are all her to listen to you, this way they will accept you more as a person and accept the way you look and we can become friends, this way the sick children will not feel ashamed anymore….and this way bullying will decrease.*

Ronaldo, a 17-year-old adolescent suggested that the app should be secured by a password:

*It is better to have a password to access the app, this will keep what we say to each other confidential and not disseminated to others.*

### A 3D approach

All participants distinctly described the concept of having a 3-dimensional approach without explicitly mentioning the term.

Queen, a 12-year-old girl stated:

*I would like to see my friends’ faces on the app while chatting with them and feel as if I am sitting with them.*

Princess, a 13-year-old girl mentioned along the same line:

*It is nice if we can see the person we are talking to and feel as if we are sitting together in the same room and also to see his/her facial expressions when in pain, this way we will feel each other’s pain….we can talk out our feelings and amuse ourselves….Also, I would like other patients to be available whenever I feel like talking rather than having to agree on dates/times.*

Zouzou, a 16-year-old adolescent shared her concern with regard to the live chatting option through a camera:

*I would like to have the option of voice recording in the app beside the camera. I am not ready at all times to be on the camera. Sometimes I do not feel like wearing my clothes or in good mood to be on camera. Sending a voice message or recording a message would be a good alternative and it is more comfortable.*

### Fear of the unfamiliar

Participants shared valid concerns that may prevent them from using the application. Barriers could be grouped into two categories related to human and technology factors.

### Human-factor barriers

Human-factor barriers were mainly related to the feelings of disease related shame. Additionally, the apprehension of not being understood by others is another barrier listed by all participants.

Yaldiz, a 12-year-old girl mentioned:

*Some people may feel ashamed of their disease and of the way they look. They are also afraid of bullying. That is why they will not be interested in this app and they will not download it”.*

Ronaldo, a 17-year-old adolescent said:

*If the group is not interacting or if someone logs in to talk and no one answers then he/she is likely to delete the app and to not be interested to join anymore.*

May, a 12-year-old girl stated:

*I will delete the app if other children are making fun of me because of my sickness and are not nice to me.*

### Technology-factor barrier

The barriers related to technology were related to the language used and to the size and ease-of-use of the application. All participants wanted an application that could be direct, easy to use and in Arabic.

Ali, a 17-year-old adolescent said:

*I want the app to be in Arabic not in English, I do not understand English.*

Also, Bob a 12-year-old adolescent stated:

*I want the app to be in Arabic and those who are going to be on the app to talk in Arabic, I barely read Arabic I won’t understand French or English.*

In parallel, Superman, a 14-year-old adolescent said:

*I want the app to be in my native language, Arabic.*

Yaldiz, a 12-year-old girl proposed an idea:

*The app could be multilingual because some patients do not understand Arabic, or it could have a translation option.*

Almost all participants mentioned that they will not download the app if it takes too much phone memory. For example, Yaldiz, a 12-year-old girl stated:

*I won’t download such an app if it takes too much space on the cellphone and if it requires Wi-Fi, some people do not have a Wi-Fi source*.

Also, Bob, a 12-year-old boy said:

*I will not download the app if it is fake, it might damage my cell phone also, if it takes too much memory.*

Hamada, a 16-year-old adolescent stated:

*I will not download the app if it takes too much memory.*

Hamada, a 16-year-old adolescent stated:

*I will not download the app if it is complicated to use. It has to be easy for all children to use it and should not take too much phone memory.”*

Ronaldo, a 17-year-old adolescent said:

*The app should be easy for use. If it is complicated, children will hate to open it and use it. For example, it has to be multilingual so children can choose the language they speak and understand.*

Maya, a 12-year-old girl said:

*I will download the app if it is easy to use and not complicated.*

Ali, a 17-year-old adolescent mentioned:

*If it is an easy app., I will download it. I mean not complicated because I do not know how to use the cell phone well and it has to be in Arabic.*

## Discussion

This study sheds light on the potential use of a new approach for pain management in children and adolescents with cancer within the Lebanese culture. The acceptability to connect through an application is supported by responses provided by Lebanese children and adolescents with cancer during face-to-face interviews. The participants agreed that having a mean to socially and emotionally connect with other cancer patients may help them in their experience of pain. This may be due to the fact that in chronic medical conditions like cancer, physical and psychological issues become increasingly interrelated, which can explain how a psychological intervention, such as social support, may help in the management of a physical symptom, like pain [71, 72]. It seems, however, that it might not apply to all stages of a person’s diagnosis. Hauken and Larsen (2019) found that in the early stages of treatment of young adult cancer patients, participants were not interested to receive support from their cancer peers because they did not want to identify themselves as one. However, as treatment progressed, this need became paramount for most participants, and those who didn’t have the chance to meet cancer peers in person sought to establish such connections online [36]. Hauken and Larsen (2019) also identified that social support was found to be perceived as beneficial only if it was unconditional and given with empathy, which goes in-line with the findings of this study.

Furthermore, participants expressed their need to be distracted from their pain, and to feel supported and heard by likeminded individuals. Not to mention that the participants were excited about having a playful virtual presence that can help them maneuver their treatment journey, which is in line with findings of previous research that highlighted the importance of connectedness for adolescent patients with cancer [59, 73, 74]. It is worth noting that our participants belong to generation GEN Z. This generation grew up within a culture of “ Always on” technological environment since they were born (https://www.pewresearch.org/fact-tank/2019/01/17/where-millennials-end-and-generation-z-begins accessed March 28, 2023). which justifies well the use of e-health. For instance, participants expressed the desire to have a 3-D interface through which they could interact with other patients and simulate reality in these interactions, and they described a setting where each participant has their own avatar that is constantly available in the application. These findings can be attributed to the interactive aspect of social support, 3D interface, and avatar interventions, which are attractive to children and adolescents, catching their attention and making them more involved in the management of their pain [75] ; they can also be attributed to the positive effect of new technologies, especially virtual reality and robotics, on the management of pain, particularly as effective means of distraction [72].

In addition to studying the impact of social support on pain management in children and adolescents with cancer, this study aimed to determine what factors facilitate or hinder the use of pain management mobile applications in this population. Research supports studying such factors in the context of mHealth applications, as it is necessary to know which elements of these applications would improve usability and best promote pain management [56]. To focus on facilitating factors, a major component that supports the use of pain management applications is the benefits they pose for patients outside the healthcare setting, as detailed by the participants in this study and in adjacent research [45]. This is due to the fact that mobile applications provide patients with the ability to communicate and manage their symptoms in real-time, a characteristic that has been proven to significantly improve children and adolescents with cancer experience of pain [75, 76]. Other facilitating factors identified in the literature include an intervention based on well-established health behavior and communication theory, and appropriate human-computer interface [77]. These factors achieved the best results in inducing behavioral change in the target population [77]. Thus, mobile applications adhering to the aforementioned factors are seen as practical and more likely to be encouraged by both patients and health care professionals [73, 78].

On the other hand, the following items were identified as negatively affecting the usability of the application by the participants of this study: ease of use, language settings, application size, and Wi-Fi accessibility. Other studies have identified the same factors and more, including developmental age of the user, high costs, and cultural factors [72, 75, 79–81]. Interestingly, the fear of being pitied was also a main concern for the participants; they insisted on connecting only with other children and adolescents who are living the same experience. The fear of being pitied, or possibly bullied, has been established as a common concern for cancer patients in Lebanon [82]. In fact, pity and bullying are well described behavioral patterns inflicted by society on cancer patients identified in other research [83, 84]. A systematic review by Collins et al. (2019) identified bullying as a significant problem for many childhood cancer patients and survivors, compromising patients’ recovery journey and inducing a myriad of academic, social and emotional challenges. Moreover, Zeighami Mohammadi et al. (2018) indicated that pity caused negative emotional reactions and forced cancer patients to resort to self-protection strategies such as social isolation. This indicates a need to consider all factors that can compromise the effectiveness of a pain management application, particularly one dealing with social support, during the development stage of the application, for serious concerns about usability and comfort might arise if not managed early on.

With that being said, it is key to keep in mind that such applications must be designed to tailor to the target population, and must be delivered with customizable and continuously accessible evidence based interventions [78]. In this way, developers can create applications that are culturally acceptable and can address the unique concerns of the target population. In addition, application developers whose main intervention is social support must bear in mind that bullying and fear are serious concerns that should be addressed prior to launching the application. With these considerations in mind, our proposed application will be tailored to our population of interest, i.e. Lebanese adolescents with cancer, and can be later on tweaked to better fit other populations. The app’s active population will be limited to the cancer patients who would be able to create and customize their personal avatar first to ensure patients’ privacy and second to improve adherence. Virtual forums will be monitored and moderated by healthcare professionals with a certification in counseling and access to psychological counseling will also be provided in group settings and for individual patients who seeks it. Through positive reinforcement, patients will be encouraged to discuss this treatment journey and success stories can be highlighted to create a sense of optimism and hope within the app’s community. The application moderator will be professionally equipped to correct misleading information and answer patient’s concerns.

Moreover, although present research supports the benefits of using social support interventions in chronic illnesses such as HIV, adult cancer, and mental illness [85, 86], research focusing on the effect of social support interventions in pediatric cancer patients are scarce. Thus, future research can focus on studying the effect of social support intervention on pain management in this specific population to better understand the benefits and drawbacks it may have. This calls for a bigger role to be played by nurses as patient educators, advocates, and caregivers.

### Practice implications

Nurses can play a key role in teaching children and adolescents with cancer about the significance of social support and assisting them to establish their social support network. Children and adolescents with cancer need to voice out their need for help. Our proposed application can be used to record pain and receive treatment related information, this is but one aspect of the what this proposed application would provide. We are considering a more holistic approach to pain management where social support is the centerpiece complemented by expert psychological support and counseling. The interaction on the application could be managed by any health care professional that will have the privilege to correct any misleading shared information.

### Limitations

Limitations of the study include the sampling process whereby the sample might not accurately represent the larger population of adolescent. Only participants willing to share their ideas and thoughts were asked to participate. However, what is needed in the current study is the depth of information rather than its generalizability. Generalizability is not a goal of qualitative studies, but rather transferability. The experience stays private, but its connotation and significance become public [87]. The goal of this study is to assess the acceptance of children and adolescents with cancer and of the use of an application for pain evaluation.

## Conclusion

This study is the first to assess the acceptability of a mobile health app for supportive pain management in children and adolescents with cancer in Lebanon. This study through the creation of such an application represents a preliminary step towards introducing technology in the pain assessment and management of adolescents with cancer in Lebanon. Findings from this study attested to the acceptability of such applications by our sample. Moreover, the overall feel and benefit of using this approach was assessed. From a technical point of view, this first phase of the project provided insights on the desired features and elements that would make introducing social technology into pain management a successful endeavor. Based on the outcome of this study, the ultimate goal (to be addressed in future research) is to develop a pain management paradigm powered by social support accessible through a smartphone app. The app will provide a parallel complementary pain management tool over existing pain treatment.

## Data Availability

The data sets used and/or analyzed during the current study available from the corresponding author on reasonable request.
